# Supplementary figures and images for: Deep neural networks for endemic measles dynamics: Comparative analysis and integration with mechanistic models
Source: PLoS Comput Biol. 2024 Nov 21;20(11):e1012616. doi: 10.1371/journal.pcbi.1012616 (PMC11620694; doi:10.1371/journal.pcbi.1012616)

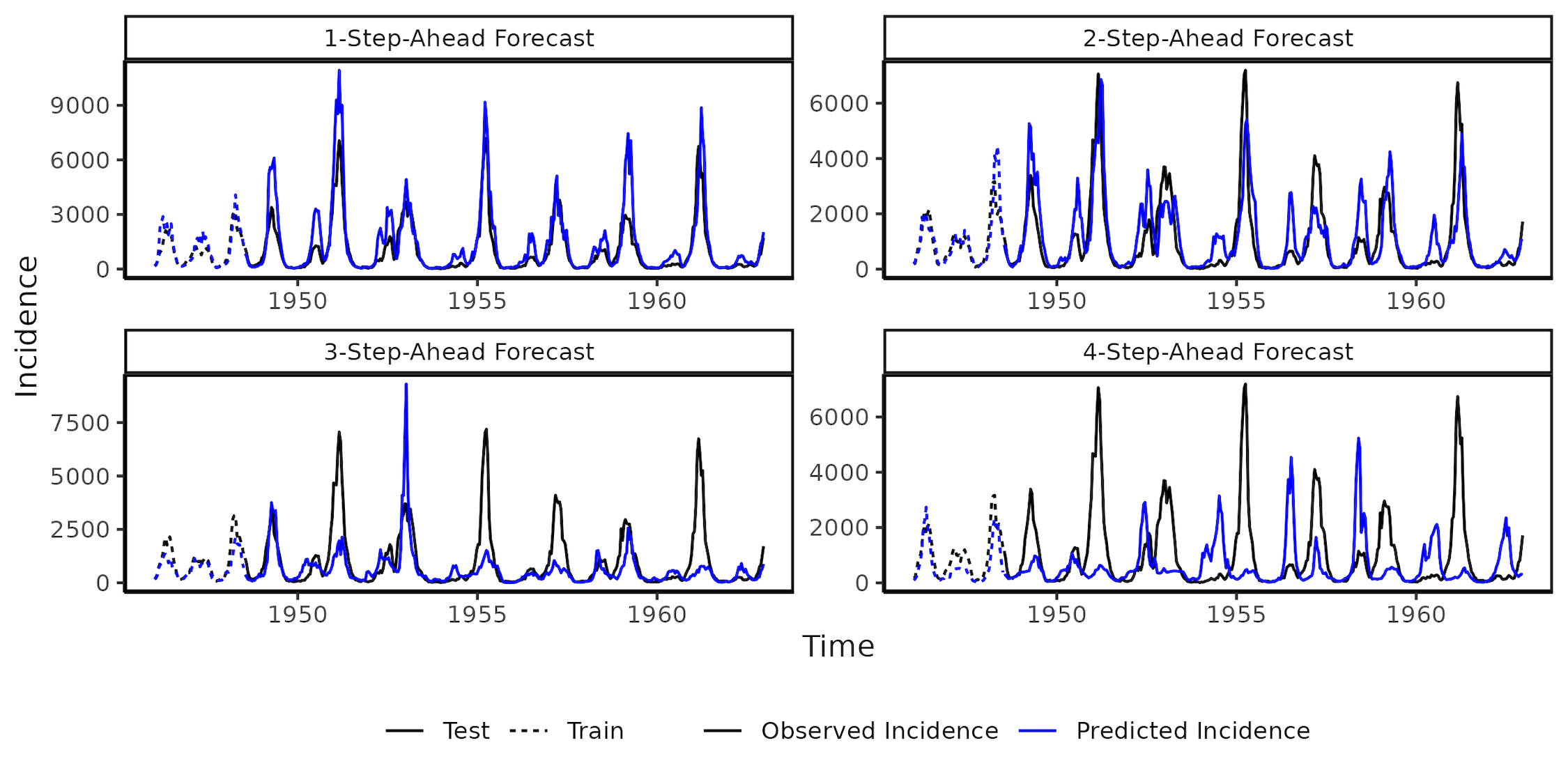

Supplement: S1 Fig — Our SFFN model trained on the limited data prior to 1948 predicts change of seasonality (i.e., annual to biennial bifurcation in late 1940s) in London, for steps-ahead ranging from 1–4. It is noted that, due to the lack of training data in this case, our SFNN does not perform well in capturing the magnitude of the incidence in general. (TIFF) [file pcbi.1012616.s001.tiff]
