# Supplementary material for: Deep neural networks for endemic measles dynamics: Comparative analysis and integration with mechanistic models
Source: PLoS Comput Biol. 2024 Nov 21;20(11):e1012616. doi: 10.1371/journal.pcbi.1012616 (PMC11620694; doi:10.1371/journal.pcbi.1012616)
Supplement: S1 Table — The average within-city standardized test-set RMSE for TSIR and SFNN for all combinations of k forecasting windows ∈ {1, 4, 12, 20, 34, 52} and train/test cutoff years ∈ {1960, 1961, 1962} demonstrates that the improvement of the SFNN model over the TSIR is stable across train/test cutoff points. (PDF) [file pcbi.1012616.s002.pdf]

Table S1. Forecasting year train/test cutoff sensitivity analysis. The average within-city standardized test-set RMSE for TSIR and SFNN for all combinations of k forecasting windows $\in \{1, 4, 12, 20, 34, 52\}$ and train/test cutoff years $\in \{1960, 1961, 1962\}$ demonstrates that the improvement of the SFNN model over the TSIR is stable across train/test cutoff points.

**k**	**Train/Test Cutoff Year**	**Test RMSE**$_{TSIR}$	**Test RMSE**$_{SFNN}$
1	1960	0.8274	0.6025
1	1961	0.8349	0.6102
1	1962	0.9004	0.6516
4	1960	1.1528	0.8053
4	1961	1.1614	0.8090
4	1962	1.2349	0.8779
12	1960	1.3395	0.8786
12	1961	1.3435	0.8703
12	1962	1.3843	0.9278
20	1960	1.3296	0.8745
20	1961	1.3324	0.8905
20	1962	1.3548	0.9341
34	1960	1.2595	0.8860
34	1961	1.2784	0.9185
34	1962	1.2654	0.9236
52	1960	1.1745	0.8853
52	1961	1.1742	0.8640
52	1962	1.2168	0.9125
