# Supplementary material for: Deep neural networks for endemic measles dynamics: Comparative analysis and integration with mechanistic models
Source: PLoS Comput Biol. 2024 Nov 21;20(11):e1012616. doi: 10.1371/journal.pcbi.1012616 (PMC11620694; doi:10.1371/journal.pcbi.1012616)
Supplement: S2 Table — Tuned hyperparameter values, as determined by Ray Tune grid search, indicate the optimal number of incidence feature time lags, hidden dimension, and weight decay value, for each forecasting window. (PDF) [file pcbi.1012616.s003.pdf]

Table S2. Optimal SFNN hyperparameters. Tuned hyperparameter values, as determined by Ray Tune grid search, indicate the optimal number of incidence feature time lags, hidden dimension, and weight decay value, for each forecasting window.

k	T_{lag}	Hidden Dimension	Hidden Layers	Weight Decay
1	26	240	1	0.0027
4	78	240	1	0.0011
12	78	1201	1	0.0103
20	78	1201	3	0.0009
34	104	1201	1	0.0135
52	130	721	2	0.0096
